# The Effects of CFTR and Mucoid Phenotype on Susceptibility and Innate Immune Responses in a Mouse Model of Pneumococcal Lung Disease

**DOI:** 10.1371/journal.pone.0140335

**Published:** 2015-10-15

**Authors:** Evida A. Dennis, Mamie T. Coats, Sarah E. Griffin, Joanetha Y. Hale, Lea Novak, David E. Briles, Marilyn J. Crain

**Affiliations:** 1 Department of Microbiology, University of Alabama at Birmingham, Birmingham, Alabama, United States of America; 2 Department of Biological Sciences, Alabama State University, Montgomery, Alabama, United States of America; 3 Department of Anatomic Pathology, University of Alabama at Birmingham, Birmingham, Alabama, United States of America; 4 Department of Pediatrics, University of Alabama at Birmingham, Birmingham, Alabama, United States of America; Centers for Disease Control & Prevention, UNITED STATES

## Abstract

Recent studies have reported the isolation of highly mucoid serotype 3 *Streptococcus pneumoniae* (*Sp)* from the respiratory tracts of children with cystic fibrosis (CF). Whether these highly mucoid *Sp* contribute to, or are associated with, respiratory failure among patients with CF remains unknown. Other mucoid bacteria, predominately *Pseudomonas aeruginosa*, are associated with CF respiratory decline. We used a mouse model of CF to study pneumococcal pneumonia with highly mucoid serotype 3 and non-mucoid serotype 19A *Sp* isolates. We investigated susceptibility to infection, survival, and bacterial counts from bronchoaviolar lavage samples and lung homogenates, as well as associated inflammatory cytokines at the site of infection, and lung pathology. Congenic CFTR^–/–^ mice and wild-type (WT)-mice were infected intranasally with CHB756, CHB1126, and WU2 (highly mucoid capsular serotype 3, intermediately mucoid serotype 3, and less mucoid serotype 3, respectively), or CHB1058 (non-mucoid serotype 19A). BAL, lung homogenates, and blood were collected from mice 5 days post-infection. Higher CFU recovery and shorter survival were observed following infection of CFTR^–/–^ mice with CHB756 compared to infection with CHB1126, WU2, or CHB1058 (*P≤0*.*001*). Additionally, CFTR^–/–^ mice infected with CHB756 and CHB1126 were more susceptible to infection than WT-mice (*P≤0*.*05*). Between CFTR^–/–^ mice and WT-mice, no significant differences in TNF-*α*, CXCL1/KC concentrations, or lung histopathology were observed. Our results indicate that highly mucoid type 3 *Sp* causes more severe lung disease than non-mucoid *Sp*, and does so more readily in the lungs of CFTR^–/–^ than WT-mice.

## Introduction

*Streptococcus pneumoniae* (*Sp*) is a leading cause of morbidity and mortality worldwide, especially in children, the elderly, and those with underlying diseases. *Sp* generally colonizes the upper respiratory tract asymptomatically, but can also cause serious invasive disease including complicated pneumonia, bacteremia, and meningitis [1]. *Sp* has been reported in the sputum of CF patients in a few previous studies, although little is known about its specific role in cystic fibrosis (CF) lung disease [2–6]. CF is a recessive genetic disorder caused by mutation in the cystic fibrosis transmembrane conductance regulator (*CFTR*) gene [7]. Approximately 30,000 children and adults in the US and 70,000 worldwide are currently affected by CF, with 1,000 new cases diagnosed each year in the US [8]. Defects within *CFTR* can lead to the build-up of abnormally thick mucus that can obstruct the airways and provide a favorable environment for bacterial infection [9–10]. Pulmonary infection with an exaggerated inflammatory response is a major cause of morbidity and mortality in CF [11]. CF patients often fail to eradicate bacteria despite a high influx of neutrophils, and it is thought that the resultant inflammation plays a major role in the deterioration of lung function, and in subsequent morbidity and mortality [11– 12].

Acquisition of mucoid *Pseudomonas aeuginosa* (*Pa)* in the lung has been correlated with decline of lung function in CF patients [13] and with transition to a highly mucoid phenotype during the course of CF disease, which is associated with a poor outcome in CF patients [9, 14–20]. When examining a prospective collection of pediatric *Sp* isolates isolated in Birmingham, Al, between 2003–2010, we noted that a significant number of respiratory pneumococcal isolates were from CF patients. The most common serotypes identified were 3 and 19A [Coats et al. in preparation]. 19A isolates, which always have a relatively non-mucoid phenotype on blood agar, were common among sputum samples from patients with and without CF. In sharp contrast, serotype 3 isolates with their mucoid phenotype on blood agar, were disproportionately represented in CF sputum, compared to their low frequency in respiratory specimens from patients without CF [Coats et al. in preparation]. In contrast to *Pa*, the clinical significance of *Sp* with different amounts of mucoid capsule has not been well defined in CF lung disease.

In the studies described here, we compared aspiration pneumonia with *Sp* of capsular serotypes 3 and 19A in mice with and without a *CFTR* defect. We compared the effects of capsule type and amount of capsule on mouse survival, pro-inflammatory cytokines TNF- α and CXCL1/KC production, lung histology, and on numbers of recovered bacteria from BAL fluid, lung homogenates and blood. The CFTR^–/–^ mice used in this study were unique in that they had a secondary mutation, insertion of human CFTR (*h*CFTR) in intestinal epithelial cells, thus permitting the mice to be nutritionally healthy enough for studies of lung infection [21]. Our findings suggest that defects in *CFTR* may increase susceptibility of mice to lung infections with the highly mucoid serotype 3 *Sp* despite similar lung histopathology and levels of TNF- α and CXCL1/KC produced early in infection.

## Materials and Methods

### Bacterial strains and growth conditions

*Sp* strains CHB756 and CHB1126 (serotype 3) and CHB1058 (serotype 19A) were all isolated as part of routine clinical care of CF patients under a protocol approved by the UAB Institutional Review Board. Cultures were stored at -80°C in 10% glycerol. Strain WU2 (serotype 3) was originally obtained from a dental patient in the 1970s, mouse passaged several times to maintain virulence in mice [22], before being stored as a single stock in 1980 at -80°C in 10% glycerol for the last 30 years. *Sp* were recovered by scraping this frozen stock with a sterile applicator and inoculating Todd-Hewitt Broth containing 0.5% yeast extract (THY). The *Sp* were grown to an optical density of 0.4–0.5 at 600 nm, aliquotted into freezer vials, and stored in THY containing 10% glycerol at -80°C until use.

### Capsule quantitation

Capsular polysaccharide was quantified among serotype 3 strains by measuring acidic polysaccharides with Stains-All 1-Ethyl-2-[3-(1-ethylnaphtho[1,2-d]thiazolin-2-ylidene)-2-methylpropenyl]naphtho[1,2-d]thiazolium bromide, 3,3′-Diethyl-9-methyl-4,5,4′,5′-dibenzothiacarbocyanine (Sigma Aldrich, St. Louis, MO) as previously described [23]. Capsule carbohydrate for type 3 strains was standardized relative to that of WU2.

### Mice

Male and female CFTRm^*1UNC−/−*^ (FABP-hCFTR) mice, (referred to as CFTR^–/–^) and CFTRm^*1UNC+/+*^ (FABP-hCFTR) [referred to as wild type (WT)] were obtained from the Gregory Fleming James Cystic Fibrosis Research Center at UAB. The intestinal abnormalities, commonly seen in mice lacking systemic CFTR-dependent chloride transport were corrected by insertion of human *CFTR* (*hCFTR*) expressed from a rat intestinal fatty acid-binding protein (FABP) promoter in the intestinal epithelial cells, allowing long-term survival of CFTR^–/–^ mice without a specialized diet [21]. CFTRm^*1UNC+/+*^ (FABP-hCFTR) mice were crossed with CFTRm^*1UNC+/-*^ (FABP-hCFTR) mice in order to get CFTR^–/–^ mice, and CFTRm^*1UNC+/+*^ (FABP-hCFTR) mice were crossed with CFTRm^*1UNC+/+*^ (FABP-hCFTR) mice to get WT-mice.

### Lung Infections

CFTR^–/–^ and WT-mice were anesthetized lightly with isoflurane. Following anesthesia, suspensions of approximately 1x10^5^ (low dose) or 7x10^**5**^ (high dose) CFU bacteria in 40μL of lactated Ringer's solution were introduced into the nares of the mice to induce aspiration pneumonia. Five days later, mice were bled retro-orbitally under isoflurane anesthesia and then euthanized using CO_2_ narcosis. Following sacrifice, the tracheas were intubated and the lungs were lavaged once with 1 mL of lactated Ringer's to collect BAL fluid. Next, lungs were harvested and homogenized in 1 mL of lactated Ringer's. The BAL fluids, lung homogenates, and blood were plated in serial threefold dilutions onto blood agar supplemented with gentamicin (4μg/mL) and incubated overnight at 37°C with 5% CO_2_ before colonies were counted. Colony counts were expressed as CFU per lung, CFU per total BAL fluid, and CFU per ml of blood [24].

### Cytokines

To examine the cytokine response early in infection, CFTR^–/–^ and WT-mice were intra-nasally infected with suspensions of 1x10^5^ CFU bacteria in 40μL lactated Ringer's solution and sacrificed 24 hours post-infection. Lung tissue and BAL were collected as previously described. Bacteria from each tissue were quantified by plating onto blood agar, and levels of TNF-α (eBioscience, San Diego, CA) and CXCL1/KC **(**R&D Systems Inc., Minneapolis, MN) were determined using commercially available ELISA kits according to the manufacturer’s instructions.

### Lung Histology

After 24 hour infection with CHB756 or WU2, lungs were collected, fixed in 4% paraformaldehyde–phosphate-buffered saline, sectioned, and stained with hematoxylin and eosin using standard techniques as described previously [25]. The degree of inflammation in mouse lungs was blindly graded microscopically as 0, 1, 2, or 3 by a pathologist (LN); grade 0 represents no inflammatory infiltrate in the tissue, grade 1 shows rare inflammatory cells, grade 2 reveals small aggregates of inflammatory cells, and grade 3 is characterized by diffuse inflammatory infiltrate.

### Statistical analysis

**Fig 1:**
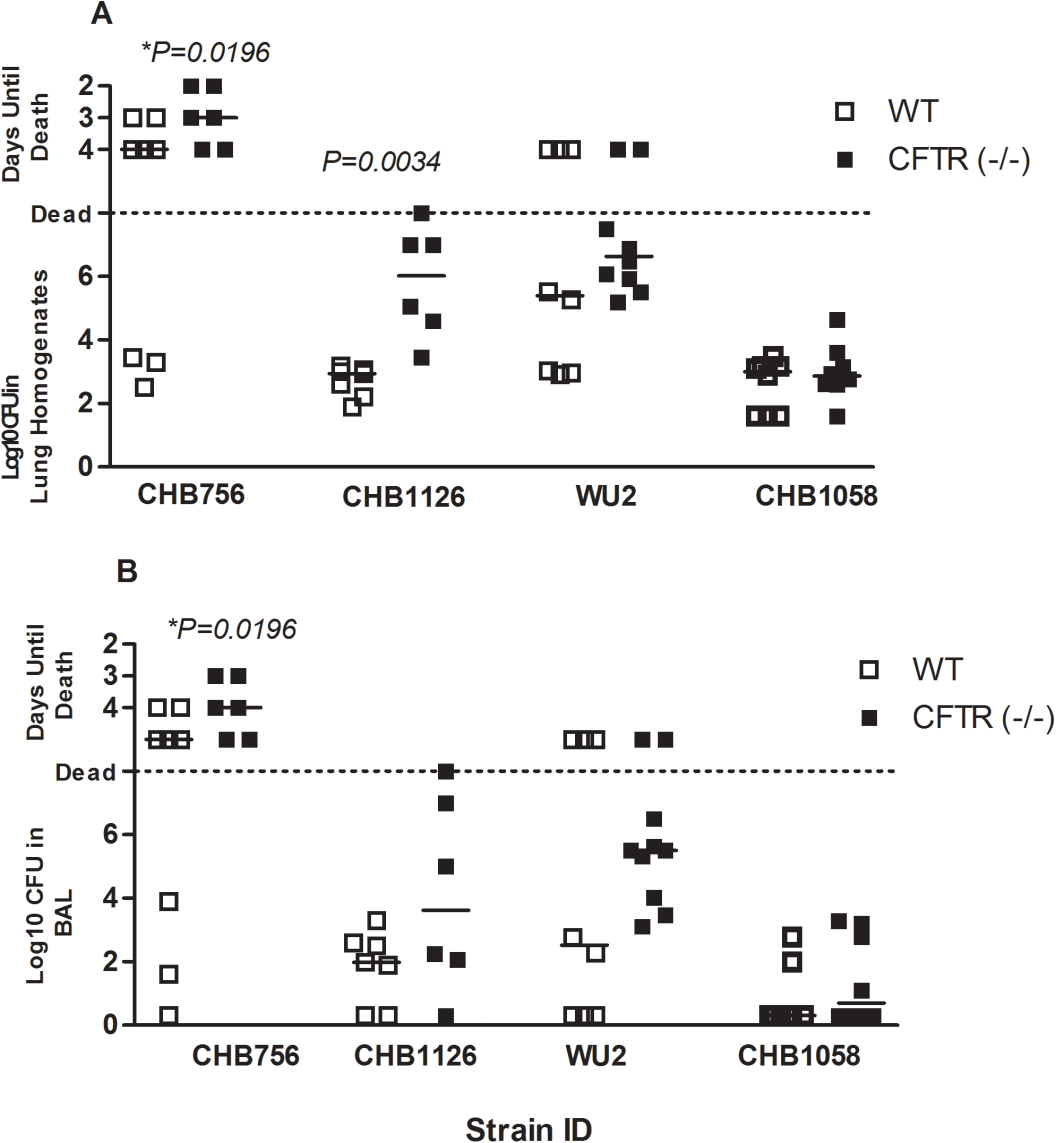
The effect of *CFTR* on bacterial clearance. CFTR^–/–^ mice (solid boxes) and WT- mice (open boxes) were infected with 7x10^5^ CFU of *Sp* strain CHB756, CHB1058, or WU2 (n = 8–10) or 1x10^5^ CFU of *Sp* strain CHB1126 (n = 6). CFUs were quantified from lung homogenates (A) and BAL fluid (B) 5 days post-infection. Each data point represents one mouse. The horizontal lines indicate the median for each group. CFTR^–/–^ mice and WT-mice were directly compared for each strain using the Mann- Whitney (two-tailed) Test on ranked data (as described in the methods). *P-values* are for comparisons between WT and CFTR^–/–^ mice in each treatment group, Experiments were repeated at least twice. *P-values ≥ 0*.*05* are not shown. Statistical comparisons between results with different challenge strains are listed in Table 2.

**Fig 2:**
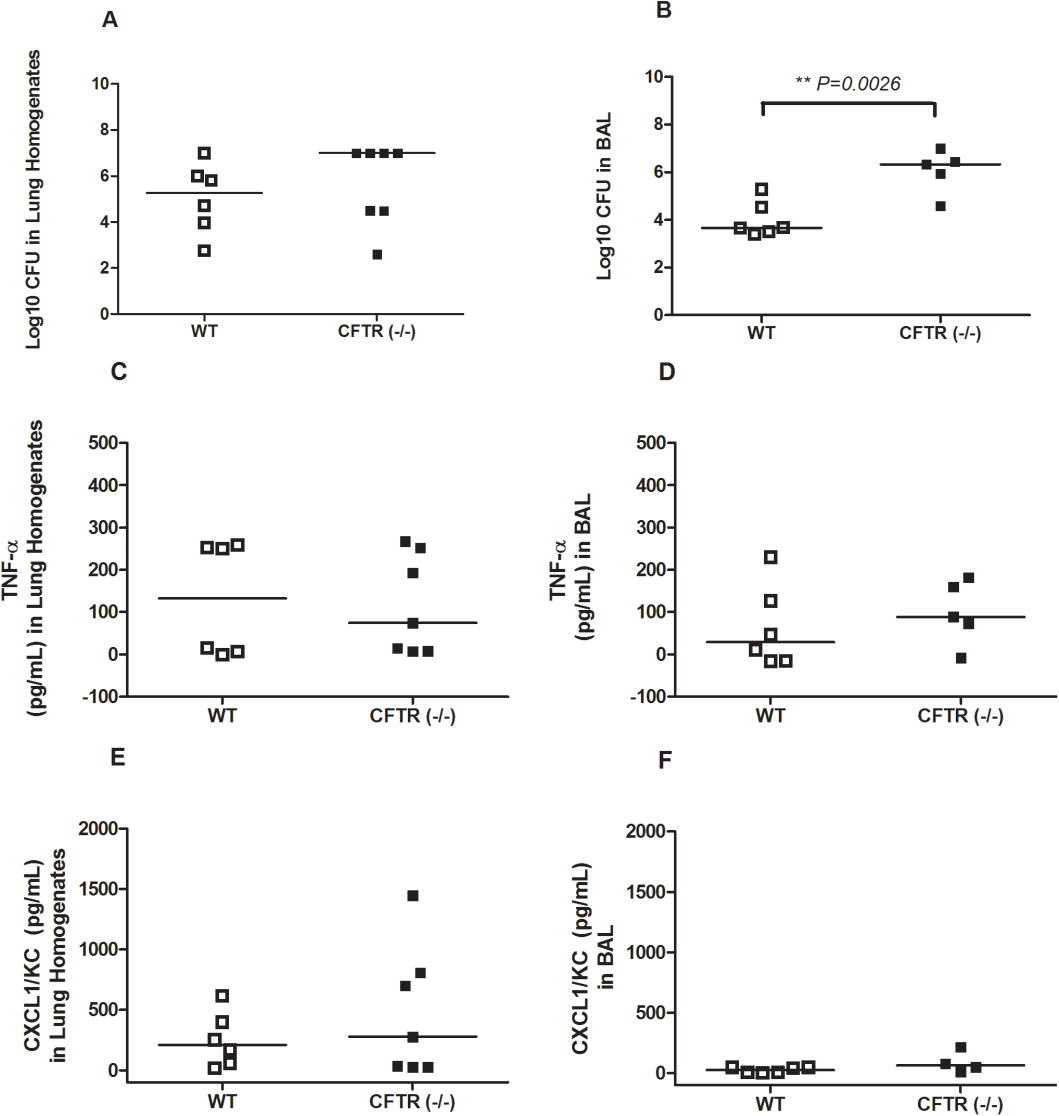
Cytokine production after intranasal infection with mucoid *Sp* isolate CHB756. CFTR^–/–^ mice and WT-mice were infected with 1x10^5^ CFU of bacteria (n = 6–7). CFUs were quantified from lung homogenates (A) and BAL fluid (B) 24 hours post-infection. TNF-α production was measured by ELISA in lung homogenates (C) in BAL (D). CXCL1/KC production was measured by ELISA in lung homogenates (E) and in BAL (F). Each dot represents the data for one mouse. The horizontal lines indicate the median for each group. Experiments were repeated at least twice. *P-values≥ 0*.*05* are not shown.

All statistical evaluations were conducted using Graph Pad Prism software version 5.03 December, 10 2009 for Windows. In Figs 1 and 2, the horizontal line denotes the median of the observed values of bacterial counts in lung homogenates, BAL, and cytokine data. The *p*-values comparing the CFTR^–/–^ and WT-mice challenged with each bacterial strain were calculated using non-parametric rank order statistics (Mann-Whitney (two-tailed) rank test). Quantified CFU were transformed to log_10_ CFU for statistical analysis. In some groups mice died prior to day 5 when CFUs were determined. Thus, the time to moribund data were included along with the CFU data from the euthanized mice such that a higher rank consistently represented shortened survival and/or worse disease as follows: mice dying on day 5 post-infection were assigned a rank order higher than that of any mice sacrificed on day 5. Mice dying on day 4 were given a higher rank than those dying on day 5, and so on. Mice dying 1 day post-infection were assigned the highest rank of all mice in the experiment. Using these ranks for the WT and CFTR^–/–^ mice infected with each strain, the statistical comparisons were calculated using the Mann-Whitney (two-tailed) rank test. When statistical comparisons were made between infections with three challenge strains, 1-way ANOVA with Tukey's Multiple Comparison Post-test was used. Cytokine data were analyzed with the unpaired two-tailed t-test.

### Ethics Statement

This study was carried out in strict accordance with the policies and approval of the UAB Institutional Animal Care and Use Committee (IACUC) of the University of Alabama at Birmingham (UAB) (APN: 090708819). Bacterial isolates from CF patients were obtained under a protocol approved by the UAB Institutional Review Board. None of the authors have any intellectual property or non-grant compensation related to the results of these studies.

## Results

### Capsule Polysaccharide in *Streptococcus pneumoniae* Isolates used in Mouse Infections

Serotype 3 *Sp* isolate CHB756 produced 428% and CHB1126 produced 122% more polysaccharide capsule relative to WU2 (100%) (Table 1). CHB756 was designated as “highly mucoid”, CHB1126 as “intermediately mucoid”, and WU2 as “less mucoid”. These strains allowed comparison between pneumococci with different amounts of type 3 polysaccharide on the susceptibility of CFTR^–/–^ mice to mucoid *Sp* infection. The type 3 strains (CHB756, CHB1126, and WU2) all were designated as mucoid based on their highly glossy colony appearance on blood agar plates and their difficulty to manipulate with cotton swabs. The type 19A strain (CHB1058), which had a type 19A capsule, was referred to as non-mucoid and provided a comparison between a relatively non-mucoid capsular type 19A strain that was originally isolated from CF sputum and the 3 mucoid type 3 strains. Among *Sp* capsular serotypes, group 19 strains invariably exhibit very low virulence in mice when given parenterally [26–28], but when given nasally are able to cause focal pneumonia that infects the lung but does not cause bacteremia or sepsis [27].

**Table 1:** *Sp* Strains used for Mouse Infections: Capsule Quantification Relative to WU2.

Strain ID	Serotype	Capsule Quantification (%)	Colony Description
CHB756	3	428‡	Highly Mucoid
CHB1128	3	122‡	Intermediately Mucoid
WU2	3	100	Less Mucoid
CHB1058	19A	N/A*	Non-Mucoid

‡Percentages were normalized to WU2

*Because Stains-All does not give the same intensity of staining capsules of different structures, a direct comparison by Stains-All of 19A with type 3 capsule was not made.

### The Effect of *CFTR* on Mouse Susceptibility to Lung Infection

The ability of CFTR^–/–^ mice to clear lung infections of each challenge strain was directly compared to WT-mice (Fig 1). Statistical comparisons used a Mann-Whitney two-sample rank test to combine information from the time (days) to moribund state prior to 5 days post-infection and the CFUs detected at 5 days post-infection. CFTR^–/–^ mice infected with 7x10^5^ CFUs of the highly mucoid serotype 3 strain, CHB756, had significantly poorer outcomes in terms of time to moribund and higher bacterial burdens in their lung homogenates (Fig 1A) and BAL (Fig 1B) than their WT-counterparts (*P* = *0*.*0196*). In order to focus on the disease process in the lung and to minimize the risk of mice becoming moribund before the end of the 5 day infection cycle, a separate cohort of CFTR^–/–^ and WT-mice were infected with mucoid serotype 3 strain CHB 1126, which produces 122% more capsule than WU2 (Table 1), but we used a lower infectious dose of 1x10^5^. At this lower infectious dose, survival was 100% by day 5 in CFTR^–/–^ mice and WT-mice. Even so, statistically significantly more CFUs were recovered from the lungs (*P = 0*.*0034*) (Fig 1A) and slightly more CFUs were recovered from BAL (Fig 1B) of CFTR^–/–^ mice than WT-mice. This result further supports our hypothesis that CFTR^–/–^ mice have heightened susceptibility to mucoid pneumococci compared to WT-mice. There were no significant differences in results observed in lung tissue and BAL CFU when CFTR^–/–^ mice were compared to WT-mice after infection with WU2 or CHB1058, although for WU2, the median showed more severe infections in the CFTR^–/–^ mice. No *Sp* were observed in the blood of any CFTR^–/–^ or WT-mice sacrificed on day 5 (data not shown).

### The Effect of Strain and Serotype on Mouse Susceptibility to Lung Infection

Table 2 illustrates the relative susceptibility of CFTR^–/–^ and WT-mice to the high dose of *Sp* infection with CHB756, WU2, and CHB1058. Infections with the three strains in CFTR^–/–^ mice produced very different results with in both BAL and lung. The highly mucoid serotype 3 CHB756 was significantly more virulent, as measured by mouse survival time and CFU recovered, than the laboratory serotype 3 strain WU2, which was significantly more virulent than the type 19A strain CHB1058

**Table 2:** Comparison of CFU recovered from BAL and Lung Homogenates from CFTR^–/–^ and WT-mice infected with serotype 3 and 19A isolates of *SP* from CF patients.

Mice	Sample	Comparison			*P-value*
CFTR^–/–^	Lung Homogenate	CHB756‡	versus	WU2	*<0*.*001*
		CHB756‡	versus	CHB1058	*<0*.*001*
		WU2‡	versus	CHB1058	*<0*.*001*
CFTR^–/–^	BAL	CHB756‡	versus	WU2	*<0*.*001*
		CHB756‡	versus	CHB1058	*<0*.*001*
		WU2‡	versus	CHB1058	*<0*.*001*
WT	Lung Homogenate	CHB756	versus	WU2	ns
		CHB756‡	versus	CHB1058	*<0*.*01*
		WU2‡	versus	CHB1058	*<0*.*05*
WT	BAL	CHB756	versus	WU2	ns
		CHB756‡	versus	CHB1058	*<0*.*01*
		WU2	versus	CHB1058	ns

‡ = Mouse was significantly more susceptible to strain

ns = non-significant

Differences between CHB756, WU2, and CHB1058 were analyzed by 1-way ANOVA with the Tukey’s Multiple Comparison Post-Test.

Infections with these bacteria in WT-mice did not show such marked differences between isolates of different serotypes or levels of mucoidy, in part, because none of the strains caused as much disease in WT-mice as in the CFTR^–/–^ mice. Even so, CHB756 was statistically more virulent in WT-mice than CHB1058 when the combination of the survival data and CFU in either the BAL fluid or lung homogenates was examined. Although WU2 again appeared to be intermediate in virulence in WT-mice and its virulence was not statistically different from either CHB756 or CHB1058 in BAL.

### The Effect of CFTR on Lung Innate Inflammatory Responses to Infection with Highly Mucoid *Sp*

To assess a potential mechanism of heightened susceptibility of CFTR^–/–^ mice to highly mucoid CHB 756, we examined CFUs recovered from BAL and lungs as well cytokine production and tissue inflammation. We used the lower infectious dose (from that used in Fig 1) of 1x10^5^ CFU to minimize risks of mice becoming moribund within the first 24 hours. CFTR^–/–^ and WT-mice were infected with highly mucoid CHB756. BAL fluids and lung homogenates were collected after 24 hours to measure cytokine production (Fig 2), and CFUs were quantified from all mice at this lower infectious dose (Fig 2A). At 24 hours post-infection, CFTR^–/–^ mice had almost 1000 fold more CFUs recovered from the BAL compared to CFU from the BAL of WT-mice (*P* = *0*.*0026*) (Fig 2B), which was also true for CFTR^–/–^ mice infected with CHB1126 (data not shown). However, in the case of the lung homogenates, although there were more median CFUs in the CFTR^–/–^ than WT homogenates, the difference was not statistically significant for infections with CHB756 (Fig 2A) or for CHB1126 (data not shown).

TNF-α and CXCL1/KC levels were quantified from lungs and BAL of the same mice examined above to assess the impact of *CFTR* on early recruitment of inflammatory mediators in the respiratory tract. These two cytokines both increase robustly in the first 24 hours in response to *Sp* infection. There were no significant differences in production of TNF-α or CXCL1/KC in the lung homogenates (Fig 2C and 2E) or the BAL (Fig 2D and 2F) between CFTR^–/–^ and WT-mice after 24 hours of infection. Additionally, we analyzed TNF-α production from CFTR^–/–^ and WT-mice infected with either highly mucoid CHB756 or non-mucoid CHB1058 at 3 hours and 6 hours post-infection. We did not see apparent differences in CFUs recovered from BAL or in TNF-α in BAL and lungs between any of the groups (data not shown) at these earlier time points. Furthermore cytokine levels were very low at these early time points.

**Fig 3:**
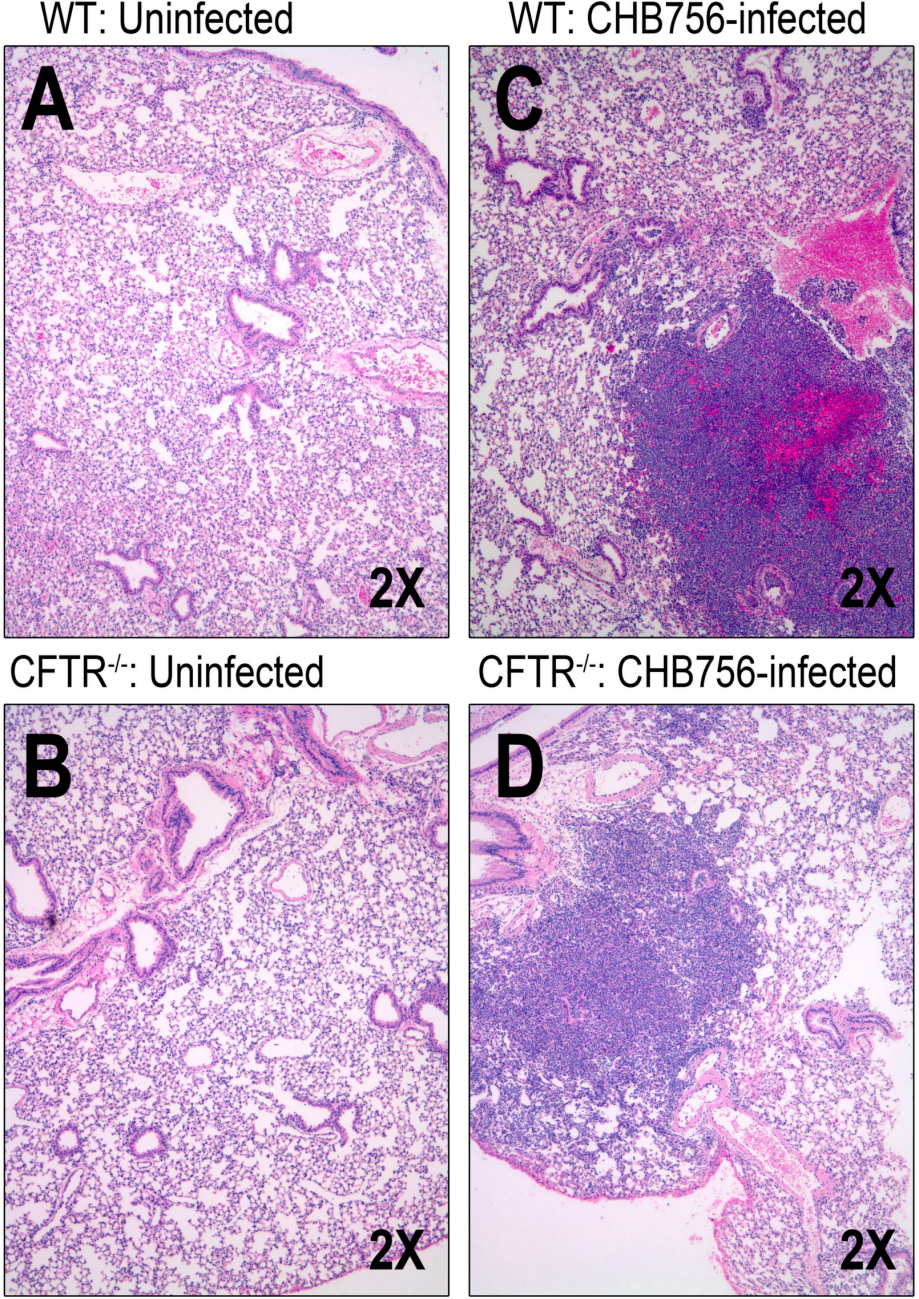
Lung histopathology after intranasal infection with mucoid *SP* isolates CHB756 and WU2. Control WT-mice (n = 2) (A) and CFTR^–/–^ mice (n = 2) (B) were left untreated. Lung histology of mucoid *Sp* infection was assessed in WT-mice (n = 2) (C) and CFTR^–/–^ mice (n = 2) (D) 24 hours after intra-nasal infection with 1x10^5^ CFU CHB756.

We selected at random 4 mice infected with CHB756 (2 CFTR^–/–^ and 2 WT), 4 infected with WU2 (2 CFTR^–/–^ and 2 WT), and 4 uninfected control mice (2 CFTR^–/–^ and 2 WT) after 24 hours to evaluate lung histopathology (Fig 3). Among uninfected mice, one WT-mouse had no inflammation (grade 0), and the other had grade 1 mild inflammation (Fig 3A). One uninfected CFTR^–/–^ mouse had grade 2 moderate inflammation at baseline; the other had grade 1 mild inflammation (Fig 3B). Among mice infected with the highly mucoid *Sp* CHB756, all CFTR^–/–^ and WT mice developed severe pneumonia, marked by diffuse inflammatory infiltrates with many neutrophils 24 hour post-infection (Fig 3C and 3D). Specifically, both CHB756-infected WT-mice lungs showed grade 3 severe inflammation in ≥ 3 lobes (Fig 3C) and lungs from both CFTR^–/–^ mice infected with CHB756 had grade 3 severe focal inflammation in a single lobe (Fig 3D). In a separate experiment with WU2, we observed much less pathology and evidence of inflammation (data not shown). These small differences in histologic inflammation in CFTR^–/–^ versus WT-mice infected with both WU2 and CHB756 were not statistically significant for either infected or non-infected mice.

Our studies have revealed a CFTR-related susceptibility to lung disease with virulent highly encapsulated pneumococci as measured by survival at 5 days, bacterial load in BAL fluids and lung tissue, and time to moribund state followed by infections with 7 x 10^5^ CFU. At a lower infection dose, we also observed CFTR-related susceptibility to lung disease with virulent highly encapsulated pneumococci measured by bacterial load in BAL fluids and lung tissue at 5 days and 24 hours post-infection. We did not however observe any statistically significant evidence for CFTR-associated inflammation in mice infected with 10^5^ CFU after 24 hours, despite higher recovery of CFUs from CFTR^–/–^ deficient mice compared to WT-mice. In our infection model, only the highly and intermediately mucoid type 3 strains caused more severe infections in CFTR^–/–^ than in WT mice. Thus, it appears that the apparent susceptibility of CFTR^–/–^ mice to mucoid pneumococcal infection is dependent on degree of mucoidy as measured by polysaccharide, but not on early recruitment of inflammatory cytokines.

## Discussion

In CF disease, chronic infection is associated with deterioration of lung function and is the major cause of morbidity and mortality. Although it is clear that the fundamental cause of CF is the lack of functional CFTR protein, the mechanisms leading to chronic lung disease associated with infection need further study. Gut-corrected *CFTR* knockout mice have provided models to assess the effects of the CF mutation on the respiratory tract [21], and were utilized in this study. The observation that mucoid *Sp* were isolated from the sputum of CF patients more frequently than from patients without CF [Coats et al. in preparation] prompted the question of whether mice with a *CFTR* defect are more susceptible to infection with highly mucoid *Sp* compared to their congenic WT littermates since other mucoid bacteria, most notably *Pseudomonas aeruginosa (Pa)*, are associated with progression of CF lung disease [9, 14–19, 29–31].

We have demonstrated more severe pneumococcal lung disease in an aspiration pneumonia disease model in CFTR^–/–^ mice compared to WT-mice infected with a highly mucoid type 3 *Sp* strain isolated from a CF patient (CHB756) and an intermediately mucoid type 3 *Sp* strain isolated from a CF patient (CHB1126). Other pneumococcal strains (a less mucoid type 3 strain, WU2, and a non-mucoid type 19A, CHB1058), were relatively avirulent in both CF and WT-mice. It should be noted that in less resistant strains of mice WU2 can cause disease even by the intravenous route [22]. These results showed that mucoid capsular serotype 3 *Sp* could exploit the CF defect of the CFTR^–/–^ mice. Lack of *CFTR* in transgenic CF mice has similarly been shown to lead to increased levels of *Pa* in the lung which are associated with hypersusceptibility of CF mice to mucoid *Pa* infection [32, 33]. Although our results support the association of a highly mucoid *Sp* with poor prognosis in CFTR^–/–^ mice, it is not clear why mice with this mutation are less able than WT-mice to clear mucoid *Sp* from their lungs. The recruitment of neutrophils to sites of infection was demonstrated histologically to be similar in the WT and CFTR^–/–^ mice and there was no difference in pro-inflammatory cytokine secretion in lung homogenates and BAL.

High susceptibility to infection in CF lung disease has been attributed in part to bacterial adaptation within the CF airways, specifically to the transition of bacteria to mucoid phenotypes such as *Pa*. The impaired ability of CF airway cells to mobilize secretions due to increased mucous production and reduced ciliary movement may favor the adherence of certain bacterial species and drive shifts to mucoid phenotypes. Mucoid *Pa* in the CF lung has been specifically associated with compromised bacterial clearance [34–36] increases in resistance to phagocytosis, antibiotics [15, 19–20] and with biofilm formation [13–14] compared to non-mucoid *Pa*, indicating that mucoidy may be an adaptive mechanism of the bacteria to permit prolonged survival especially within the CF host. Our observations that mucoid type 3 *Sp* isolates appear more frequently in the lungs of CF patients than in normal individuals, and that CFTR^–/–^ mice are more susceptible to highly mucoid *Sp* than WT-mice, both support the notion that mucoid pneumococci may have an advantage in the environment of the CF lung which may lead to chronic infection.

Heightened bacterial susceptibility in CF lungs has also been credited to changes in the host that result in an impaired ability to regulate immune responses [11, 37–38]. Inflammation has been shown to be present early in the course of CF lung disease even prior to infection [39]. Pro-inflammatory cytokines and neutrophil markers in children with CF are elevated compared to children without CF [40–41], and infection with pathogenic bacteria have been demonstrated to exacerbate secretion of pro-inflammatory cytokines by CF airway cells [12]. Additionally *CFTR* has been shown to regulate the functions of circulating and resident cells involved in immune surveillance, and dysfunction of *CFTR* directly alters innate immune responses [34, 37]. In our lung histopathology studies, uninfected CFTR^–/–^ mice actually had higher levels of baseline inflammation compared to uninfected WT-mice (Fig 3C and 3D), thus a failure to make an inflammatory response does not seem to be the mechanism of susceptibility to pneumonia with highly mucoid pneumococci in the *CFTR* deficient mouse model.

In this study, differences in TNF- α, CXCL1/KC, and lung histology were compared between CFTR^–/–^ mice and WT-mice after 24 hours of infection with highly mucoid *Sp* strain CHB756. TNF- α was selected for measurement because this cytokine is produced into the alveolar space within a few hours following lung infection of mice with many bacterial species including *Pa* [42], *Staphylococcus aureus* [43], and *Sp* [44], and is essential for initial bacterial defense. Furthermore, following *Sp* infection, TNF- α initiates a wave of cytokine production that affects the adaptive immune response [44]. The mouse IL-8 homologue, CXCL1/KC, was also selected for measurement. Previous studies have suggested that prolonged neutrophilia associated with continuous production of CXCL1/KC in mice may allow increased airway damage from neutrophil products and provides a favorable setting for further bacterial invasion [34, 45]. Despite higher susceptibility of CFTR^–/–^ mice to CHB756 when compared to congenic WT littermates, the production of TNF-α and CXCL1/KC were only slightly, but not significantly higher in CFTR^–/–^ mice than in WT-mice. These results suggest that the CF genetic background in mice may not significantly affect cytokine production and neutrophil recruitment during the first 24 hours of infection with highly mucoid *Sp*. Differences in susceptibility between CFTR^–/–^ and WT-mice do not appear to be dependent on these cytokines or necessarily effect production of these cytokines. Future studies should include analysis of biofilm formation by type 3 pneumococci under *in vitro* and *in vivo* conditions as a potential mechanism of increased virulence and persistence by highly mucoid strains.

We have studied infections with mucoid and non- mucoid *Sp* in an animal model of CF lung disease. Because mucoid phenotype may protect organisms from clearance from the CF lung, it may exacerbate the cycle of chronic infection and exaggerated inflammation. Advancing our knowledge of the microbiology of CF lung disease, pathogenesis, and subsequent immune responses should improve the prospect for better therapies aimed to reduce inflammatory deterioration, regulate immune responses, and to effectively reduce the disease burden cause by highly mucoid bacteria.
